# Microbial electron transport and energy conservation – the foundation for optimizing bioelectrochemical systems

**DOI:** 10.3389/fmicb.2015.00575

**Published:** 2015-06-11

**Authors:** Frauke Kracke, Igor Vassilev, Jens O. Krömer

**Affiliations:** ^1^Centre for Microbial Electrochemical Systems, The University of Queensland, BrisbaneQLD, Australia; ^2^Advanced Water Management Centre, The University of Queensland, BrisbaneQLD, Australia

**Keywords:** microbial electron transport, redox potential, microbial electrosynthesis, microbial fuel cell, bioelectrochemical system, bio electrochemistry, acetogenic bacteria, ATP yield

## Abstract

Microbial electrochemical techniques describe a variety of emerging technologies that use electrode–bacteria interactions for biotechnology applications including the production of electricity, waste and wastewater treatment, bioremediation and the production of valuable products. Central in each application is the ability of the microbial catalyst to interact with external electron acceptors and/or donors and its metabolic properties that enable the combination of electron transport and carbon metabolism. And here also lies the key challenge. A wide range of microbes has been discovered to be able to exchange electrons with solid surfaces or mediators but only a few have been studied in depth. Especially electron transfer mechanisms from cathodes towards the microbial organism are poorly understood but are essential for many applications such as microbial electrosynthesis. We analyze the different electron transport chains that nature offers for organisms such as metal respiring bacteria and acetogens, but also standard biotechnological organisms currently used in bio-production. Special focus lies on the essential connection of redox and energy metabolism, which is often ignored when studying bioelectrochemical systems. The possibility of extracellular electron exchange at different points in each organism is discussed regarding required redox potentials and effect on cellular redox and energy levels. Key compounds such as electron carriers (e.g., cytochromes, ferredoxin, quinones, flavins) are identified and analyzed regarding their possible role in electrode–microbe interactions. This work summarizes our current knowledge on electron transport processes and uses a theoretical approach to predict the impact of different modes of transfer on the energy metabolism. As such it adds an important piece of fundamental understanding of microbial electron transport possibilities to the research community and will help to optimize and advance bioelectrochemical techniques.

## Introduction

The fact that some bacteria are able to transport electrons beyond their cell wall and therefore electrically interact with their environment is known for over a century (Potter, 1911). This feature can be used to develop advanced electrically enhanced bio-processes. In so called BESs the organisms interact with electrodes via the exchange of electrons, which are either supplied or removed through an external electrical circuit. The application possibilities for electrode–bacteria interactions include the production of electricity, waste and wastewater treatment, bioremediation and the production of valuable products therefore opening a wide research field (Franks, 2012; Lovley, 2012; Yang et al., 2012; Harnisch et al., 2014). Initial research mainly focused on the application of BESs for power production. In so called microbial fuel cells microbes donate electrons to electrodes and therefore generate an electrical current (Rabaey et al., 2004; Franks and Nevin, 2010; Janicek et al., 2014). Another application is presented by bioremediation of aquatic sediments and groundwater where metal-reducing microbes catalyze the transformation of organic contaminants to carbon dioxide (Williams et al., 2009; Zhang et al., 2010). Within these systems the anodic oxidation by bacteria is coupled to production of chemicals on the cathode, usually hydrogen or methane, and they are referred to as microbial electrolysis cells (Wagner et al., 2009).

In recent years another approach gets more and more attention which intends a shift in the current: the technique termed microbial electrosynthesis refers the microbial production of multi-carbon compounds in a BES under current consumption. Novel sophisticated techniques like wind turbines and photovoltaic cells enable sustainable and cheap energy production and therefore allow bringing energy consuming technologies into focus (Lovley and Nevin, 2013). Initially the term microbial electrosynthesis was used exclusively for the microbial reduction of carbon dioxide with the help of electricity (Nevin et al., 2010; Rabaey and Rozendal, 2010). But the research field was quickly widened by multiple studies that follow the same approach of optimizing microbial production by electrical enhancement from other substrates than CO_2_, often referred to as electro fermentation (Kim and Kim, 1988; Emde and Schink, 1990; Shin et al., 2002; Steinbusch et al., 2009; Rabaey and Rozendal, 2010; Choi et al., 2012). Common in all studies is the aim of overcoming metabolic redox limitations by electron exchange between microbes and electrodes for increased production. In a recent metabolic modeling study, it was shown that not necessarily the degree of reduction of a product but rather the metabolic pathway that leads from the chosen substrate to the target compound decides if there is an electron surplus or demand inside the metabolic network of a cell. This was highlighted with the production of 1,4-butanediol and 2,3-butanediol from sugar. Both products have the same degree of reduction, but the different metabolic pathways employed for production will lead to different by-products and a different overall electron balance, such that in case of 2,3-butanediol a surplus of electrons needs to be balanced, while in the 1,4-butanediol case a shortage of electron limits the theoretical yield (Kracke and Krömer, 2014). Therefore cathodic as well as anodic BES are of interest and we suggest extending the term microbial electrosynthesis to all processes that intend an electrically induced shift of carbon flow toward production of valuable chemicals.

Whether one is considering current consuming or current producing bio-processes, crucial in each application is the performance of the microbial host. The ability and especially the efficiency of the organism to exchange electrons with an electrode and connect this EET to its cmetabolism significantly influences the overall process performance. Many applications in BESs are so far restricted to lab-scale research projects as the electron transfer rates are simply too low to design a viable process scale-up (Logan and Rabaey, 2012). In order to optimize and advance bioelectrochemical techniques a more thorough understanding of possible extracellular electron exchange mechanisms in both directions is needed (Tremblay and Zhang, 2015). Especially cathodic systems and the fundamentals of electron transport towards microbes are poorly understood (Rosenbaum et al., 2011; Sydow et al., 2014). In this article we discuss the natural electron transport chains of different organisms and discuss their potential benefits and limitations if used in a BES. Special focus lies on the connection of redox and energy metabolism in each species.

## Varieties of Microbial Electron Transport Chains

In microbial electron transport chains electrons are transferred from a low potential electron donor to an acceptor with more positive redox potential by redox reactions. These reactions are usually catalyzed by membrane-bound compounds that use the energy difference between donor and acceptor to establish an ion-gradient across the membrane, which in turn is used for ATP synthesis and thus converts the difference in electrical potential into chemical energy for the cell (Anraku, 1988).

In order to adapt to different environmental conditions microbes developed an enormous variety of electron transport chains (Hernandez and Newman, 2001). Important systems catalyzing these redox reactions include primary dehydrogenases that supply high energetic electrons from a donor such as NADH and usually couple electron transport to H^+^ or Na^+^ transport across the membrane (Anraku, 1988). Also involved in transmembrane ion-transport are membrane-localized (multi-) protein complexes such as cytochromes and terminal oxidases (reductases) that transfer electrons to a final acceptor such as oxygen, nitrate or fumarate (Hannemann et al., 2007; Vignais and Billoud, 2007; Richter et al., 2012). Most transmembrane reductases and oxidases function as ion pumps but some do not. Electron carrying co-factors such as quinones, flavines, heme, iron–sulfur clusters or copper ions also play an important role in microbial electron transport. Some of these are soluble lipophilic molecules that shuttle electrons between the relatively large enzymatic complexes inside the membrane (e.g., quinones) while others are catalytic cofactors bound to proteins (e.g., heme groups of cytochromes; Deller et al., 2008; Dolin, 2012). There are also membrane bound complexes that use the exergonic electron bifurcation of soluble cellular redox compounds such as ferredoxin (Fd) and NADH for transmembrane ion transport and therefore establishment of a motive force across the membrane that can drive ATP synthesis (Herrmann et al., 2008; Müller et al., 2008; Schuchmann and Müller, 2012).

The achievable energy gain (Gibbs free energy, ΔG) of each electron transport chain is depending on the redox potential difference (ΔE) of all reactions between electron donor and acceptor. Some bacteria incorporate several electron transport chains, which they can use sometimes even simultaneously in order to respond to different electron acceptors and donors available in the environment (Haddock and Schairer, 1973; Anraku, 1988). Others are restricted to only one respiratory pathway (Das and Ljungdahl, 2003; Müller et al., 2008). This diversity of microbial electron transport mechanisms illustrates the complexity of the approach of bioelectrochemical techniques. In order to interfere efficiently with the redox metabolism of an organism one needs to understand the targeted site of EET and its metabolic impact.

A wide range of microbes has been discovered to be able to exchange electrons with solid surfaces (direct EET) and/or soluble mediators (indirect EET) but only a few have been studied in depth. In fact the mechanisms of electron transport that are found in different species can differ significantly from one another. Dissimilatory metal reducing bacteria are amongst the most studied being, able to “respire” insoluble metals in anaerobic environments. The model organisms *Geobacter sulfurreducens* and *Shewanella oneidensis* were studied by various research groups for decades and evidence for direct and indirect electron transfer between the organism and electrodes could be found (Bond and Lovley, 2003; Thrash and Coates, 2008; Rosenbaum et al., 2011; Ross et al., 2011). For both bacteria outer-membrane cytochromes were identified as essential compounds to enable EET (Mehta et al., 2005; Shi et al., 2007, 2009; Breuer et al., 2015). However, there are several differences in their electron transport chains, for example *Shewanella* excretes soluble electron carriers while similar compounds are missing in *Geobacter* sp. (Holmes et al., 2006; Marsili et al., 2008a). Furthermore it could be shown that the redox chains catalyzing an inward current rely on different mechanisms than current producing reactions (Dumas et al., 2008; Strycharz et al., 2008). Another group of dissimilatory metal reducing bacteria is presented by the obligate anaerobe *Thermincola*, which were also found to be capable of directly transferring electrodes to anodes (Marshall and May, 2009; Wrighton et al., 2011). Their EET mechanisms also seem to rely on cytochromes that in this case are cell-wall associated of the Gram-positive bacteria (Carlson et al., 2012). Interestingly there are also organisms such as *Clostridium ljungdahlii*, which do not have any cytochromes but were tested positive on EET (Köpke et al., 2010; Nevin et al., 2011). The exact mechanisms by which electrons are transferred between the electrode surface and the microbial metabolism still remain unclear (Patil et al., 2012; Sydow et al., 2014). Therefore we have a look at the native electron transport chains of several organisms that were studied in BESs. An overview is given in **Table 1** and the following sections discuss the mechanisms of the presented bacteria in detail.

**Table 1:** Electron transport characteristics and behavior in bioelectrochemical systems of organisms discussed in this study.

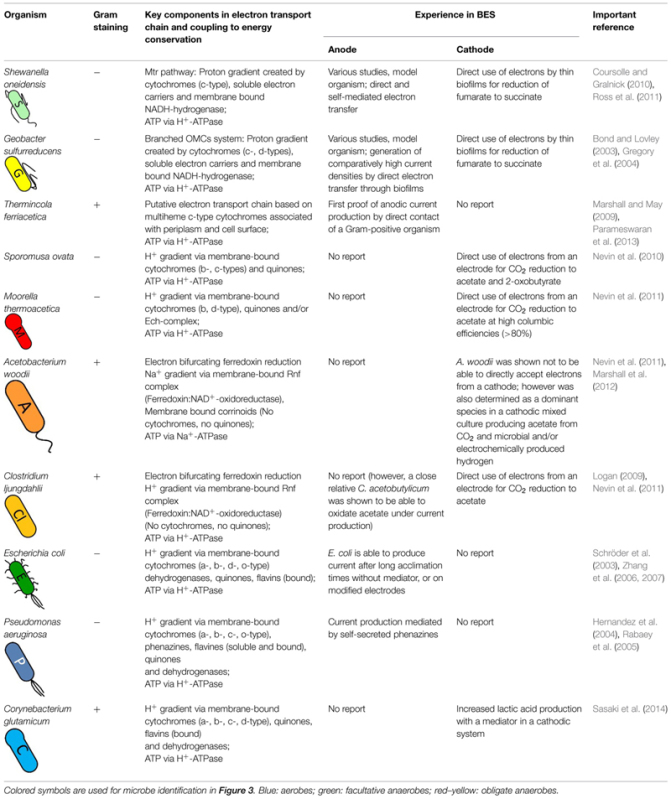

### Specific EET in Metal Respiring Bacteria

The first microorganisms that we want to introduce and discuss belong to the group of dissimilatory metal reducing bacteria. As their name indicates, the microorganisms have the ability to interact with solid minerals, e.g., Fe(III) or Mn(IV), to obtain energy by using those minerals as electron acceptors and/or donors for their respiration process. Here a transport of electrons from a low redox potential donor to an acceptor with a higher redox potential can result in a proton gradient to drive ATP synthesis. That characteristic of breathing metals plays an important role in biogeochemical cycles and has the potential to be used in bioremediation and BES (Lovley et al., 2004; Richardson et al., 2012).

But how does the microorganisms respire insoluble metals and extract energy? This question has not been fully clarified in detail yet. In contrast to other respiration processes, where freely diffusible gas or readily soluble substances can easily enter the cell and be used as electron acceptors/donors, the major challenge for metal respiring bacteria is the interaction with the extracellular minerals, which cannot pass the cell membrane and do not have access to the periplasm nor cytoplasm. To overcome this barrier the bacteria need redox-active molecules in their outer membrane or have to excrete redox-active shuttle molecules to transfer electrons between the cellular interior and the extracellular metals (Hernandez and Newman, 2001; Hartshorne et al., 2009). There exists a great diversity of mechanisms for such electron-shuttling pathways, which is explained in more detail in the section below by describing the EET of two model organisms.

#### *G. sulfurreducens*:Branched OMCs System

The Gram-negative obligate anaerobic δ-proteobacterium *G. sulfurreducens* is used as a model organism for investigating electroactive microorganisms (Levar et al., 2012). Since its genome was sequenced it is easier to analyze the detailed molecular mechanism of EET and to construct molecular models. In its genome more than 110 genes coding for putative c-type cytochromes have been identified, which likely play an important role in the electron transport pathway of this bacterium (Methe et al., 2003). It is assumed that several multiheme c-type cytochromes enable the transport of redox equivalents between the cellular menaquinone (MQ) pool and the extracellular insoluble metals to create a proton gradient for energy conservation (Levar et al., 2012). The interaction between the cytochrome complexes in the electron transport chain is based on the redox potential of the different multiheme molecules of the cytochromes, whereby each heme has its own specific redox potential. In this way wide windows of potential ranges are created that overlap with each other and allow a bio-energetic transfer of electrons (Inoue et al., 2010; Qian et al., 2011).

**FIGURE 1:**
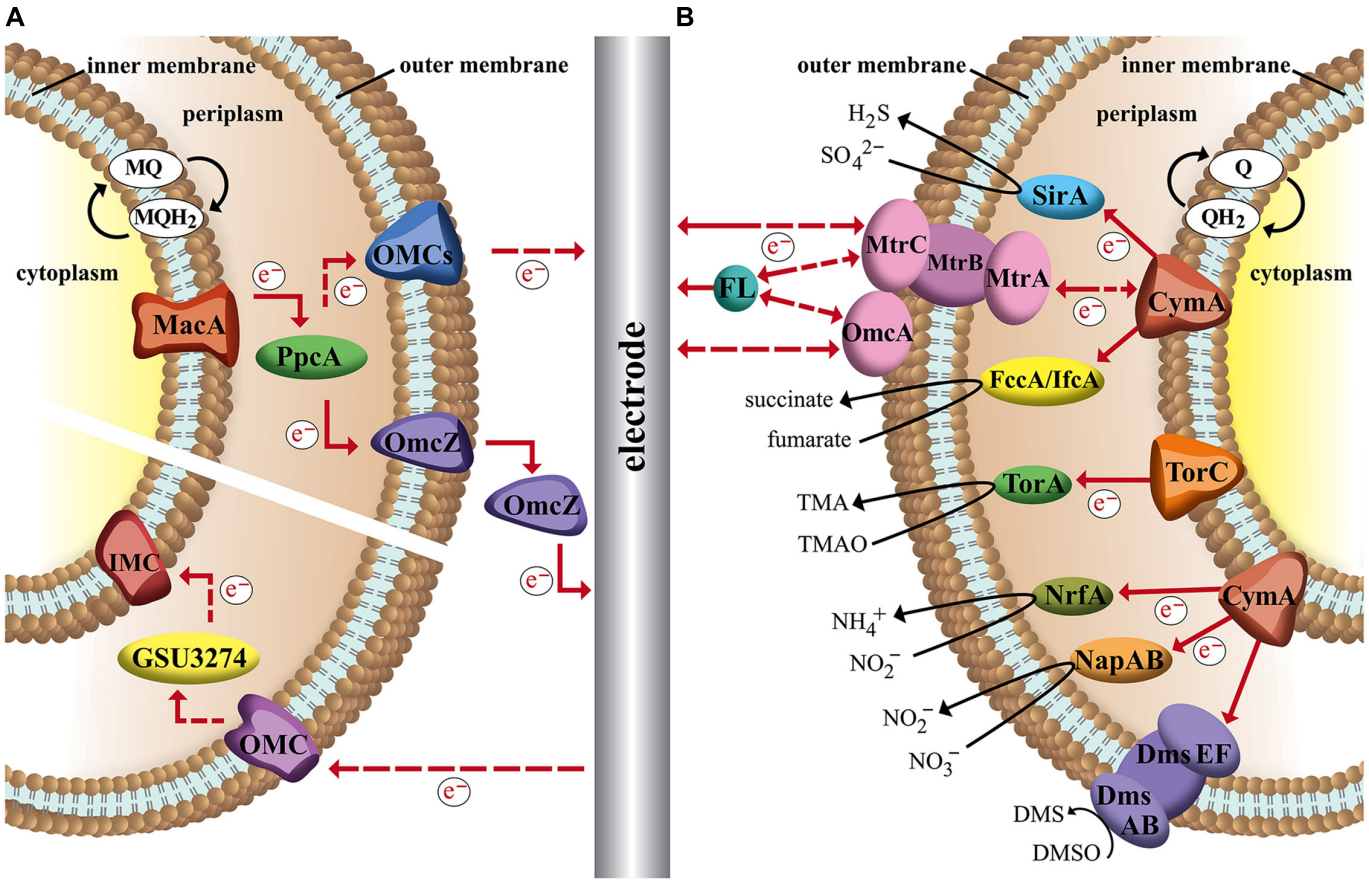
**Schematic image of the proposed EET of two metal respiring bacteria and their interactions with an electrode in a bioelectrochemical system.** Dashed arrows indicate hypothetical electron flow and solid arrows indicate experimental proved electron flow. **(A)** Branched outer membrane cytochromes (OMCs) system of *Geobacter sulfurreducens*. Electrons can be transported between inner membrane, periplasm, outer membrane, and an electrode via a chain of cytochromes and menaquinones (MQ). Terminal OMCs can vary depending on the environmental conditions. **(B)** Unique Mtr-pathway and terminal reductases of *Shewanella oneidensis*. Quinones (Q) pass electrons to CymA or TorC, which transfer the electrons to terminal reductases or a MtrCAB complex. MtrCAB complex can interact with the electrode direct or via flavin molecules (FL).

**Figure 1A** illustrates a model of the EET mechanism with the participating proteins, which were assigned a central role. Here a diheme cytochrome c peroxidase, designated ‘metal-reduction-associated cytochrome’ (MacA), functions as a transmitter of electrons from the inner membrane to the triheme periplasmic c-type cytochrome (PpcA) in the periplasm. Following on PpcA passes the electrons to the outer membrane cytochromes, termed OMCs (e.g., OmcB, OmcC, OmcS, OmcZ), which transfer the electrons to the extracellular acceptor. The branched OMCs system is very complex and is still not fully understood. It seems that different OMCs are required to interact with different extracellular metals or electrodes (Levar et al., 2012). For example, the octaheme cytochrome OmcZ is more abundant in biofilms grown on an electrode and a deletion of *omcZ* gene leads to current decrease of more than 90% while there is no impact on the reduction of other electron acceptors as Fe(III) oxide (Nevin et al., 2009; Richter et al., 2009). The dodecaheme cytochrome OmcB and the hexaheme cytochrome OmcS are essential for Fe(III) reduction while knock-out of the *omcB* or *omcS* genes results in no or hardly any effect on current generation through biofilms grown on an anode (Kim et al., 2008; Richter et al., 2012). The proposed model reflects a greatly simplified EET mechanism. In fact the EET pathway is subjected to a complex regulatory mechanism and there exist many more homologous *omc* genes, which can replace lacking cytochromes genes in generated mutants (Kim et al., 2006; Schrott et al., 2011). For instance OmcB is important for the reduction of soluble Fe(III), but an Δ*omcB* mutant can express homologs such as a dodecaheme OmcC, which allows the use of a parallel pathway to respire Fe(III) (Leang and Lovley, 2005).

In comparison to the transport of electrons to the anode, much less is known about the mechanism by which the cell takes up electrons from the cathode. Due to the reverse electron chain pathway the practicing shuttle molecules have to operate at different redox potentials. In contrast to current-generating biofilms, in current-consuming biofilms a lower expression of OMCs such as OmcZ were detected. Deletion of those associated genes, which were essential for anodic biofilms, had no impact to cathodic biofilms. In further studies it was observed, that in current-consuming biofilms a gene (GSU3274) encoding a putative monoheme c-type cytochrome was strongly expressed. Mutants lacking this gene lost their ability to take up electrons from a cathode, but did not show differences in EET to an anode. So it seems that GSU3274 plays a significant role in the EET from a cathode (Rosenbaum et al., 2011; Strycharz et al., 2011).

*Geobacter sulfurreducens* shows best EET performance as a biofilm grown on the electrode based on direct electron transfer. This bacterium possesses excellent biofilm formation properties and the thickness of the biofilm is linked with the amount of generated current in a linear correlation up to a certain thickness limit (Reguera et al., 2006). The transport of electrons in a multilayer biofilm to an anode can be achieved by two combined mechanisms. One transfer way is based on secretion of non-diffusing ‘mediators’ (e.g., cytochromes such as OmcZ) into the biofilm matrix, which can act as electron shuttles (Richter et al., 2009). The second way depends on ‘nano-wires,’ which are electrically conductive appendages that enable physical connection between cells and/or cells and surface of the electrode (Reguera et al., 2006). The mechanism of the EET in such nano-wires has not been clarified yet. One model proposes that the nano-wires are pili with a metallic-like conductivity, which is based on aromatic amino acid residues within the appendages, which enable electron delocalization due to π-stacking (Malvankar et al., 2011, 2015). Another theory describes a ‘superexchange’ model, in which the electrons are ‘hopping’ along a chain of redox active proteins to the final electron acceptor (Malvankar et al., 2012; Strycharz-Glaven and Tender, 2012).

In comparison to anodic-biofilms, it was reported, that cathodic-biofilms are less-developed and much thinner (Gregory et al., 2004; Strycharz et al., 2008). The reason might be limiting redox surplus under non-autotrophic anaerobic growth conditions, which is enhanced by cathodic electron input as suggested by a recent *in silico* study (Kracke and Krömer, 2014). However, this observation needs to be studied in depth and be validated experimentally for a better understanding.

#### *S. oneidensis*: Characteristic Mtr-Pathway

The second electroactive model organism that we want to introduce and discuss its characteristic EET mechanism is the Gram-negative facultative anaerobic bacterium *S. oneidensis*. The interesting characteristic of *S. oneidensis* is its ability to utilize a great variety of inorganic and organic compounds as final electron acceptor in the absence of oxygen. As in the case for *G. sulfurreducens* that property is based on interaction of a large number of multiheme cytochromes. The sequenced genome of *S. oneidensis* shows 42 putative cytochromes, of which 80% are located in the outer membrane (Heidelberg et al., 2002; Lower et al., 2005). The first step of the electron transfer through the cell membrane is the oxidation of small electron carriers (quinols), which enable the transport of electrons between NADH-dehydrogenase and cytochromes in the inner membrane to create a proton gradient for energy conservation in form of ATP. That oxidation reaction can be catalyzed by the tetraheme cytochromes TorC and CymA, whose sequence is very similar and both are attached to the inner membrane by a single α-helix. The next link in the electron transfer chain of TorC is a periplasmic reductase TorA, which can utilize the outer membrane permeable trimethylamine *N*-oxide compound as terminal electron acceptor and reduces it to trimethoxyamphetamine (Dos Santos et al., 1998). In comparison to TorC, CymA can interact with different redox partners in the periplasm and as a consequence outer membrane-crossing molecules like sulfite can be reduced by the octaheme redox partner SirA, (Shirodkar et al., 2011) nitrite by pentaheme cytochrome NrfA, (Gao et al., 2009) nitrate by NapAB reductase (Simpson et al., 2010) and fumarate by FccA and IfcA reductase (Maier et al., 2003). Furthermore an octaheme cytochrome OTR was detected and showed *in vitro* capability of the reduction of a range of N and S oxides and oxyanions, but *in vivo* the function of OTR has not been confirmed yet (Atkinson et al., 2007).

Additionally as a metal respiring bacterium like *G. sulfurreducens*, *S. oneidensis* is able to use extracellular terminal electron acceptors, e.g., electrodes, Fe(III) and Mn(IV) (see **Figure 1B**). To overcome the outer membrane barrier the bacterium possesses a Mtr-pathway. Homologous genes for that pathway were also found in other dissimilatory metal reducing and oxidizing bacteria (Shi et al., 2012). However, *S. oneidensis* Mtr-pathway is one of the best investigated EET chains. It has been suggested that a decaheme cytochrome MtrA takes up electrons from CymA via an electron transport chain and passes them on to an extracellular decaheme cytochrome MtrC, which transfers the redox equivalents to final exogenous electron acceptors (Myers and Myers, 2000; Coursolle and Gralnick, 2010). Here MtrA, MtrC and a third element, MtrB form a complex, MtrCAB. MtrB is a porin molecule in the outer membrane and serves to organize and stabilize MtrA and MtrC to enable electron transfer. Besides MtrC a second decaheme cytochrome, OmcA was detected anchored as a lipoprotein in the outer membrane that was able to transfer electrons to exogenous electron acceptors as well (White et al., 2013; Breuer et al., 2015). In addition to the *mtrCAB* genes the genome of *S. oneidensis* encloses homologs like *mtrFDE*. The homologous cytochromes can support the EET or can partly take over the activity of the MtrCAB complex depending on the electron acceptor and the growth conditions (Coursolle and Gralnick, 2010). Likewise, the DmsABEF complex is based on homologs. Here the porin cytochrome complex DmsEF transfer the electrons to the DmsAB complex, which is then able to reduce DMSO to DMS (Gralnick et al., 2006). The periplasmic tetraheme cytochrome STC has to be mention as well, which seems to support the transfer of electrons between inner membrane and outer membrane, however, the exact mechanism is unclear (Fonseca et al., 2013). In contrast to EET to an anode, the information about taking up electrons from a cathode is limited. The interacting compounds in the cathodic process were not established yet. It was suggested due to *in vivo* studies, where electrons were provided from a graphite electrode to reduce fumarate, that an electron uptake is possible through a reverse Mtr-pathway (Ross et al., 2011).

In contrast to *G. sulfurreducens*, *S. oneidensis* shows not only the ability of direct electron transfer, but can also perform indirect electron transfer due to excretion of redox active mediators. Direct electron transfer is based on physical connection with the electrode by forming a biofilm on the surface (Marsili et al., 2008a,b) and through extensions in form of nano-wires. While *Geobacter* nano-wires are assumed to be type IV pili, for *S. oneidensis* nano-wires it was shown, that their structure is similar to outer membrane vesicles and those nano-wires can be seen as extensions of the outer membrane and periplasm that include the multiheme cytochromes responsible for EET (Pirbadian et al., 2014). In case of indirect electron transfer, *S. oneidensis* secretes flavin molecules (FL) that act as small diffusible shuttle molecules to transfer electrons between electrode and OMCs (Marsili et al., 2008a) or as bounded cofactors for OMCs (Okamoto et al., 2013). *G. sulfurreducens* is also able to produce FL, but here the flavins are preferentially bound to the OMCs and are not mobile like free shuttle units (Okamoto et al., 2014).

### Carbon Respiration of Acetogenic Bacteria

Acetogenic bacteria, short acetogens, are anaerobic organisms that are able to assimilate CO_2_ or CO via the Wood–Ljungdahl pathway, also called carbonate-respiration or acetyl–CoA pathway. This autotrophic pathway offers the possibility to develop biotechnological processes that combine the usage of cheap ubiquitous substrates (i.e., syngas) with greenhouse gas reduction and therefore makes acetogens attractive hosts for biotechnology (Köpke et al., 2010; Tracy et al., 2012). This feature put the bacteria into focus of the research community trying to establish an artificial ‘photo’synthesis process by using CO_2_ and electrons from an electrode (Nevin et al., 2010). Acetogens are also able to utilize a great variety of heterotrophic molecules such as sugars, glycerol, and cellulose, which broadens the spectrum of possible substrates to waste streams from biodiesel industry (e.g., glycerol) or dairy industry (e.g., whey) and many more (Tracy et al., 2012). The main product is usually acetate (hence the name) but acetogens also bear the feature to naturally produce a broad spectrum of other chemicals, which are of industrial use either directly or as precursors such as ethanol, butanol, 2,3-butanediol or butyrate (Tracy et al., 2012). The mayor intermediate from carbon fixation via Wood–Ljungdahl pathway is acetyl–CoA and is linked to other metabolic pathways such as Embden–Meyerhof–Parnas pathway, therefore offering a great metabolic diversity for metabolic engineering of other production pathways.

In the Wood–Ljungdahl pathway two CO_2_ molecules are merged to form one molecule Acetyl-Coenzyme-A, which is either converted to acetate or assimilated in biomass. The overall conversion of CO_2_ to acetate uses one ATP (in the step of formyltetrahydrofolate synthetase) and creates one ATP by acetate kinase reaction. Therefore no net energy gain can be achieved via substrate-level phosphorylation. Since acetogens are able to grow autotrophically, the pathway must be coupled to a chemiosmotic mechanism that provides additional energy. By calculating the Gibbs free energies of each reaction in the Wood–Ljungdahl pathway a net energy benefit of about –95 kJ/mol can be determined (Diekert and Wohlfarth, 1994). This energy could support the synthesis of 1–2 mol ATP via chemiosmosis as anaerobic bacteria require –60 to –80 kJ of free energy for the synthesis of 1 mol of ATP (Das and Ljungdahl, 2003). In recent years experimental evidence for membrane driven ATP synthesis in acetogens could be found, however, the sites and mechanisms of energy conservation differ between organisms. Over 100 different species of acetogens have been identified and despite their common feature of CO_2_ assimilation via the Wood–Ljungdahl pathway they are very diverse in terms of metabolism, phylogenetics, or preferred habitat (Drake et al., 2008). The best-studied organisms belong to the genera *Acetobacterium* and *Clostridium* and include a few species with fully available genome sequences (genome published: *Moorella thermoacetica*, *Clostridium ljungdahlii*, *Clostridium carboxidivorans*, *Eubacterium limosum;* under preparation: *Acetobacterium woodii, Clostridium aceticum*). Genetic and genomic tools are under intense development and promise fast advancing metabolic engineering platforms for acetogens (Lütke-Eversloh and Bahl, 2011; Lee et al., 2012; Schiel-Bengelsdorf and Dürre, 2012; Banerjee et al., 2014).

#### *M. thermoacetica*: Cytochromes or Ech-Complex?

**FIGURE 2:**
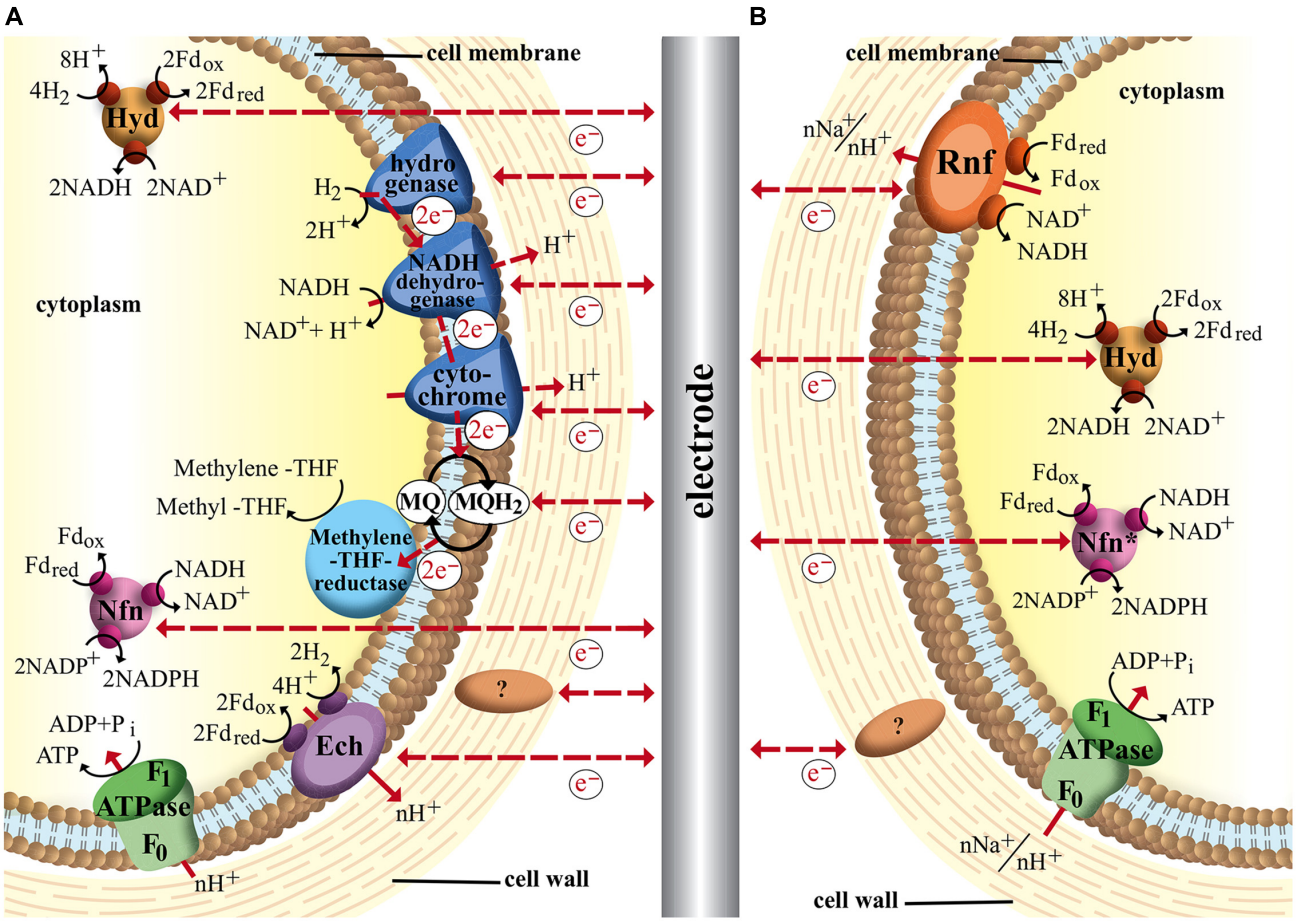
**Schematic image of acetogenic electron transport chains and possible interactions with an electrode in a bioelectrochemical system.** Dashed arrows indicate hypothetical electron and proton flow. **(A)** Electron transport mechanisms in *Moorella thermoacetica* via membrane-bound cytochromes and hydrogenases, MQ, soluble electron-bifurcating complexes (Hyd and Nfn) and proton pumping Ech-complex; **(B)** Electron transport of *Clostridium ljungdahlii* (H^+^) and *Acetobacterium woodii* (Na^+^) based on membrane-bound Rnf-complex and soluble electron-bifurcating complexes (^∗^Nfn-complex is not found in *A. woodii*). ? represents hypothetical cell-wall associated proteins that could facilitate electron transfer.

From an energetic point of view and in regards to electron-transport properties acetogens are often divided in two groups: Na^+^-gradient and H^+^-gradient dependent species. *M. thermoacetica* (formerly *Clostridium thermoaceticum*) an example for the latter group was the model organism for HG Wood and LG Ljungdahl for their studies of the acetyl–CoA pathway. They identified several membrane-integrated electron carriers MQ MK-7, a flavoprotein and two b-type cytochromes that are believed to play major parts in creating a proton gradient over the membrane (Das and Ljungdahl, 2003). Later such a proton motive force that could drive ATP synthesis in *M. thermoacetica* was measured, however, which components exactly transfer protons across the membrane remains unknown (Ragsdale and Pierce, 2008). Genome studies of the organism revealed also membrane-bound components such as hydrogenases and NADH-dehydrogenase, which are known to transfer protons across the membrane in other organisms. Therefore a membrane integrated electron transport chain via these complexes with H_2_ as electron donor and methylene-THF as electron acceptor is proposed (Müller and Frerichs, 2013). However, so far no experimental data supports this hypothesis. Recent studies also deliver evidence for electron-bifurcating enzymes that play important roles in electron transfer of autotrophic and heterotrophic pathways of *M. thermoacetica* (Huang et al., 2012). The soluble complex HydABC oxidizes hydrogen with simultaneous reduction of Fd and NAD^+^ (Wang et al., 2013). Additionally a second soluble transhydrogenase (NfnAB) catalyzes the electron bifurcation from reduced Fd and NADH to NADP^+^. Furthermore the genome of *M. thermoacetica* codes for a membrane-bound energy converting hydrogenase, called Ech-complex. For the methanogenic archaea *Methanosarcina mazei* the Ech-complex is responsible for establishment of a proton gradient across the membrane, which leads to the theory that could also be the case in acetogens (Welte et al., 2010; Schuchmann and Müller, 2014). This complex uses the excess energy that is freed from electron transfer from reduced Fd to H^+^ to transport ions across the membrane. A very recent report states that electron stoichiometry is only balanced with involvement of the Ech-complex in energy conservation while the membrane-bound dehydrogenases and cytochromes play no major part (Schuchmann and Müller, 2014). As this hypothesis also lacks distinct experimental validation we included both theories in our summary of possible electron transport mechanisms of *M. thermoacetica* (**Figure 2A**).

#### *A. woodii* and *C. ljungdahlii*: Electron Transport without Cytochromes?

Nevin et al. (2010) reported that the electrons needed for CO_2_ fixation via Wood–Ljungdahl pathway could be provided directly by an electrode, a breakthrough work in the field of bioelectrochemical techniques (Nevin et al., 2010). The acetogen *Sporomosa ovata*, a close relative to *M. thermoacetica* was able to directly accept electrons from a cathode and convert carbon dioxide to acetate and 2-oxobutyrate. Following studies showed similar abilities of other acetogens of the *Sporomosa* and *Clostridium* genera (Nevin et al., 2011). The acetogen *A. woodii*, however, was found unable to consume current and showed different behavior compared to other acetogens in Nevins experiments. *A. woodii* is an example strain for Na^+^-dependent acetogens that typically lack cytochromes. It could be shown that the conversion of CO_2_ to acetate via Wood–Ljungdahl pathway is coupled to the generation of a sodium gradient across the cytoplasmic membrane (Müller, 2003; Fast and Papoutsakis, 2012). Since *A. woodii* does not contain cytochromes or quinones energy conservation must be secured by a different electron transport system. In 2008 evidence for a novel membrane-bound Fd:NAD^+^ oxidoreductase (Rnf complex) was reported that seems to be responsible for transmembrane Na^+^ transport (Müller et al., 2008). In this complex the electrons from reduced Fd are transferred to NAD^+^ to form NADH. Since the redox potential of Fd (E^0′^_Fd_= -500 to -420 mV) is more negative than that of the NAD^+^/NADH couple (E^0′^_NADH_= –320 mV), the energy surplus (equivalent to –20 to –35 kJ/mol, three to four times more than released by the Ech-complex) is available for transmembrane ion transport (Biegel et al., 2011). This sodium gradient is harvested by the Na^+^-dependent F_1_F_0_ATP synthase of *A. woodii*. It was suggested that some steps of the Wood–Ljungdahl pathway also add to the transmembrane ion gradient. The reduction of methylene-H_4_F to methyl-H_4_F was discussed as a likely site since the reaction is exergonic and coupled to a corrinoid iron sulfur protein (Ragsdale and Pierce, 2008). But recent studies could identify the Rnf complex as the only membrane-bound electron transfer system and rather suggest a Fd reduction by the methylene-THF reductase (Poehlein et al., 2012).

Interestingly a similar Rnf complex was detected in *C. ljungdahlii* even though its membrane gradient is proton based, which would put the organism into the H^+^-acetogen-group together with *M. thermoacetica*. However, *C. ljungdahlii* does not contain any cytochromes and therefore it seems more reasonable to categorize anaerobic homoacetogenic organisms into Ech- and Rnf-containing groups with subgroups of Na^+^- and H^+^- dependent species (Köpke et al., 2010; Schuchmann and Müller, 2014). It could be shown that the Rnf complex is an electron bifurcating Fd:NAD^+^ oxidoreductase and is essential for developing a proton gradient over the membrane under autotrophic as well as heterotrophic growth conditions (Tremblay et al., 2013). With fructose as substrate and electron donor Rnf-deficient strains were growth limited with significantly reduced ATP yields as a result of disruptions in the membrane gradient development. Autotrophic growth without Rnf complex was completely inhibited, indicating the Rnf-complex being a major if not the sole electron transport mechanism linked to energy conservation. The soluble electron bifurcating complexes HydABC and NfnAB are also found in *C. ljungdahlii* while *A. woodii* is believed to only use HydABC (Schuchmann and Müller, 2014). The proposed electron transport mechanisms for both organisms are shown in **Figure 2B**.

### Other Respiratory Pathways

In the section above we introduced typical electroactive microorganisms, which were used in BES. But what about other model and/or industrial microorganisms like *Escherichia coli* and *Corynebacterium glutamicum* or pathogenic microorganisms like *Pseudomonas aeruginosa*? Can we influence/manipulate their redox and energy metabolism? In order to answer this question, it is necessary to understand their respiratory pathways.

#### *E. coli*: Model Organism with Respiratory Flexibility

The respiratory system of *E. coli* is very well known and in many studies the bacterium is used as a model for investigation of energetics and regulation of respiration. The respiratory chains show a great diversity and variability enclosing 15 primary dehydrogenases to oxidize electron donors and ten terminal reductases (or oxidases) to reduce electron acceptors (including isoenzymes; Berlyn, 1998). Those primary dehydrogenases and terminal reductases are linked by three quinones: ubiquinone (E^0′^ = +110 mV), demethyl MQ (E^0′^ = +40 mV) and MQ (E^0′^ = –80 mV). Depending on the enzymes various quantities of energy can be conserved due to the build-up of a proton gradient through proton pumps, or by arranging catalytic sites in a certain way to release the protons on opposite sides of the membrane to create a charge separation. The H^+^/e^-^ ratios vary from 0 to 4 H^+^/e^-^ (Unden and Bongaerts, 1997). Under aerobic conditions *E. coli* can conserve most energy by using quinol oxidases (E^0′^ = +820 mV) to reduce O_2_ to H_2_O. Here O_2_ represses the terminal reductases of anaerobic respiration. However, in the absence of O_2_, energy can be generated by nitrate reductases (E^0′^ = +420 mV), nitrite reductase (E^0′^ = +360 mV), DMSO reductase (E^0′^ = +160 mV), TMAO reductase (E^0′^ = +130 mV) or fumarate reductase (E^0′^ = +30 mV) with nitrate delivering the most energy and fumarate the least energy (Ingledew, 1983; Unden and Bongaerts, 1997; Berlyn, 1998). So depending on which level or in which step the electron chain is targeted or manipulated, the metabolism will gain more or less energy.

Furthermore it was demonstrated that *E. coli*, evolved under electrochemical tension in a microbial fuel cell, can generate current by using the electrode as an electron sink. It was proposed that the evolved cells possess the ability to excrete hydroquinone derivatives through a highly permeable outer membrane, which act as mediators to transport the electrons between cell and electrode (Qiao et al., 2008; Qian et al., 2011). Another approach to obtain *E. coli* cells that show electro activity is via metabolic engineering, which is discussed in Section “Key Compounds in Different Electron Transport Chains.”

#### *P. aeruginosa*: Secretion of Redox Carriers

Another interesting microorganism is the Gram-negative aerobic bacterium *P. aeruginosa*. It is an opportunistic pathogen, which can live in various environments due its ability to catabolize a large number of substances (Baltch and Smith, 1994). Additionally the bacterium is a good biofilm-builder and has branched respiratory chains to use oxygen as an electron acceptor, involving five oxidases (*bo*_3_ oxidase, *aa*_3_ oxidase, *cbb*_3_ oxidase 1, *cbb*_3_ oxidase 2, and cyanide-insensitive oxidase), which are adapted to the varying availability of oxygen in the different biofilm stages due to biofilm thickness (Jo et al., 2014). Preferentially *P. aeruginosa* obtains its energy from aerobic respiration; however, under anaerobic conditions the bacterium can also survive in presence of nitrate or nitrite by using reductases to reduce the N-molecules (Hassett et al., 2002).

When *P. aeruginosa* is cultivated in a microbial fuel cell, the bacterium shows an interesting behavior. Instead of oxygen, nitrate or nitrite *P. aeruginosa* can use the anode as an electron sink to generate energy for an active growth (Rabaey et al., 2005). The anode stimulates the production of phenazine derivatives, e.g., phenazine-1-carboxylic acid (E^0′^ = –275 mV), phenazine-1-carboxamide (E^0′^ = -150 mV) and procyanin (E^0′^ = -32 mV). The secreted phenazine derivatives operate as soluble mediators, which significantly enhance the electron transfer between electrode and cells, resulting in an increased current generation. In addition the diffusible phenazine derivatives enable the use of the electrode as an electron sink for cells in thick multilayer biofilms (Rabaey et al., 2005; Yang et al., 2012). Another attractive phenomenon; that in a mixed culture the secreted mediators can be use not only by *P. aeruginosa* itself, but also by other microorganism, which generally are not able to produce redox active mediators (Boon et al., 2008).

#### *C. glutamicum*: Oxygen Dependency

The third bacterium, *C. glutamicum*, is an important Gram-positive industrial microorganism, which has been widely used as a microbial cell factory for the production of various amino acids, nucleic acids and other chemicals in food, pharmaceutical, cosmetics and chemical industries (Tatsumi and Inui, 2012). *C. glutamicum* can utilize various carbon sources for energy conversion and oxygen as the preferable electron acceptor by using three oxidases. The *bc*_1_ oxidase can take up electrons from menaquinol and pass the electrons to the *aa*_3_ oxidase by forming a supercomplex that has a low oxygen affinity, whereas the third oxidase, a *bd* oxidase, has a high affinity to oxygen (Bott and Niebisch, 2003). The bacterium can also survive under anaerobic conditions in the presence of nitrate. However, the growth is very limited, because the bacterium has a nitrate reductase, but lacks enzymes to degrade the toxic product of the nitrate reductase (nitrite; Nishimura et al., 2007).

Manipulating the redox metabolism by cultivating *C. glutamicum* in a BES can result in higher yields of the target product (Sasaki et al., 2014). Experiments demonstrated that the bacterium grown under a cathodic potential (E^0′^ = –600 mV) using glucose as the carbon source showed a decreased growth rate and an increased lactate yield. Here the anthraquinone 2,6-disulfonate was added to the medium as an artificial mediator to shuttle electrons between cathode and cells. The mechanisms, how the redox metabolism is influenced by the artificial mediator is still unclear (Sasaki et al., 2014).

## Making the Connection: Microbe–Electrode Interaction

The previous chapter demonstrates the impressive diversity and complexity of microbial solutions for cellular electron transport. Similar to the ability to interact with many different electron donors and acceptors in the environment one can assume that microbes are also able to exchange electrons with electrodes via different cellular components and mechanisms. In order to achieve successful EET the specific characteristics of the electron transport chains of the target organism should be considered. While it is questionable if there are groups or microbes that are better adapted for EET than others it is for certain that a bio electrochemical approach is challenged by different conditions depending on the catalytic host. Organisms that feature outer membrane redox-components might be able to perform direct electron transfer with electrodes while soluble intracellular complexes such as the electron bi-furcating Nfn and Hyd complexes of acetogens are most likely only targetable via soluble mediators. For each organism the specific redox-window of its electron transport chain(s) might dictate required potentials for use in BES. The following two sections discuss key components in electron transport chains of the presented organisms and the possible metabolic impact if EET at different sites can be achieved.

### Key Compounds in Different Electron Transport Chains

**FIGURE 3:**
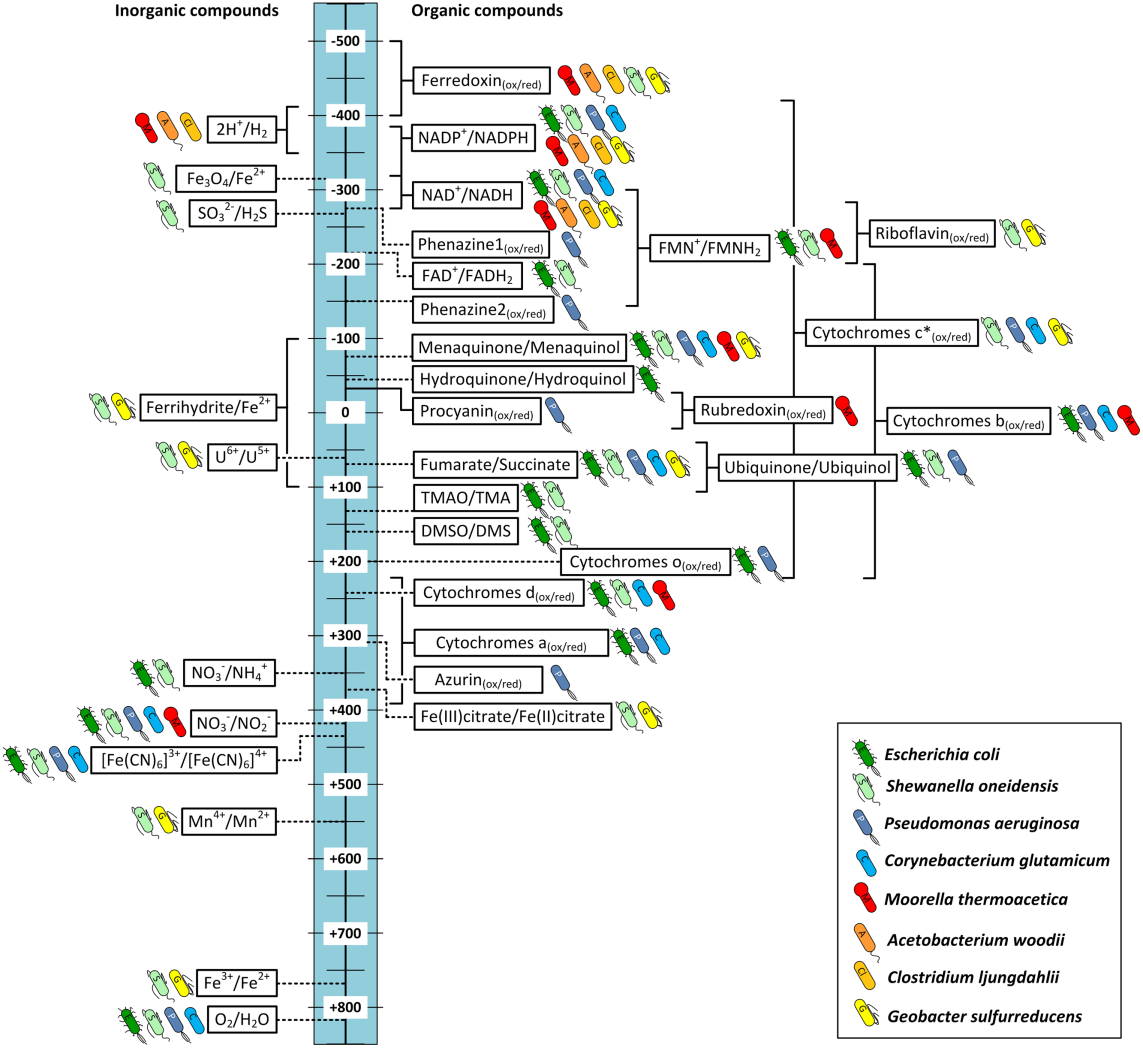
**Redox potentials of important redox reactions in electron transport chains catalyzed by the bacteria discussed in this study.** Standard redox potential (E^0′^ [mV, 25°C, pH = 7]) are indicated by dashed lines. If physiological or environmental conditions are known to shift the potential from the E^0′^, redox windows are indicated (solid lines). The bacterial symbol behind each reaction shows the organisms that are known to catalyze the reaction naturally. Blue: aerobes; green: facultative anaerobes; red–yellow: obligate anaerobes; Phenazine1 = Phenazine-1-carboxylic acid; Phenazine2 = Phenazine-1-carboxamide. ^∗^c-type cytochromes can cover a broad range of redox potentials as indicated. Not all bacteria mentioned will cover the whole range. For detailed discussion refer to main text.

With **Figure 3** we created an overview of important redox-reactions that are catalyzed by the presented organisms. Actual environmental conditions in living cells differ from the standard biochemical conditions (25°C, 1 atm, pH = 7), which might influence the actual redox potentials of each couple. The potential of the proton hydrogen couple for example is -414 mV under standard conditions. In acetogenic environments, however, this potential lies closer to –350 mV as the partial hydrogen pressure is around ∼200 Pa (Müller and Frerichs, 2013). Many intracellular redox-carriers also show different redox potential than the corresponding pure compounds. The standard redox potential of Fds with one or more iron–sulfur clusters for example lies around –400 mV (Buckel and Thauer, 2013). Under physiological conditions in acetogens, however, Fds are usually >90% reduced and therefore reported to be able to catalyze reducing reactions at redox potentials as low as –500 mV (Buckel and Thauer, 2013; Hess et al., 2013). A similar effect shifts the true redox potential of the NAD^+^/NADH couple to around –280 mV as the majority of molecules (>90%) are oxidized even though the E^0′^ under standard conditions is –320 mV. Very close to that of NADP^+^/NADPH (–324 mV), which in turn is shifted to –360 mV due to the intracellular ratio of NADP^+^/NADPH being 1/40 (Buckel and Thauer, 2013). In these cases we indicated a redox-area rather than one known midpoint potential to illustrate the potential range at which these reactions might occur inside the organisms. We allocated a fixed standard redox potential (dashed lines) or potential range for each reaction depending on availability in the literature. However the reader might want to keep in mind that environmental conditions such as pH, redox potential of the solution and specific concentrations can result in a further redox potential shift. The ion couple Fe^3+^/Fe^2+^ as an inorganic electron acceptor/donor is a good example for how the redox potential can be influenced by environmental conditions. The midpoint potential is about +770 mV at a low pH and in the absence of precipitation. However, depending on pH, concentration and in which form iron is available the midpoint potential can vary strongly (Straub et al., 2001). For example the midpoint potential of Fe_3_O_4_/Fe(II) is significantly lower (–314 mV), because magnetite is a less soluble mineral. For the organic chelate complex Fe(III)-citrate/Fe(II)-citrate the solubility is higher and, respectively, the midpoint potential (+372 mV; Thamdrup, 2000; Straub et al., 2001).

Some of the listed cellular redox compounds have been shown to play a major part in electron transfer between organisms and electrodes and therefore others might as well. As discussed above direct EET in *Geobacter* and *Shewanella* can be achieved via a network of cytochromes with different midpoint potentials [e.g., PpcA: –170 mV (Lloyd et al., 2003), OmcZ: –180 mV (Inoue et al., 2010), OmcS: -212 mV (Qian et al., 2011), CymA: -200 mV, MtrA: -100 mV, MtrC: -138 mV (Firer-Sherwood et al., 2008)]. However, for a better characterization and a functional understanding of the cytochromes their wide potential windows should be considered, because the cytochromes are complex molecules not showing narrow midpoint potentials but more wide ranges of potentials suggested to be due to the multiheme molecules in the cytochromes. Each heme has its own specific redox potential and affects the potential of the neighbor hemes creating a specific wide window of potential ranges for the overall molecule (Morgado et al., 2010; Breuer et al., 2015). For example the potential windows of OmcZ, OmcS, CymA, and MtrC are from –420 to –60 mV (Inoue et al., 2010), –360 to –40 mV (Qian et al., 2011), –0.350 to –0.080 mV and –0.280 to –0 mV (Firer-Sherwood et al., 2008), respectively. It is assumed that the wide windows allow an overlapping of the redox potentials of the cytochromes in an electron transport chain and make a thermodynamic downhill process of electron transport along the chain possible. Furthermore these wide potential ranges allow the interaction with a broad spectrum of external electron acceptors and donors (Firer-Sherwood et al., 2008; Inoue et al., 2010; Breuer et al., 2015). However, the potential windows can vary depending on the environmental conditions. For the cytochromes of *S. oneidensis* it was shown that the potential windows are shifted as a function of pH. It was observed that under higher pH conditions in most cases the redox potentials are shifted to more negative values (Firer-Sherwood et al., 2008). In **Figure 3** we allocated a broad potential range to c-type cytochromes based on the discussed literature and indicated all organisms that contain c-type cytochromes. However, this does not necessarily mean that all these bacteria are able to catalyze reactions in the indicated redox potential window. *G. sulfurreducens’* genome encodes for around 110 different c-type cytochromes (Methe et al., 2003), *S. oneidensis* for 42 (Heidelberg et al., 2002), and *P. aeruginosa* for 27 (Bertini et al., 2006), while *C. glutamicum* genome only encodes for a single c-type cytochrome (Bertini et al., 2006). Therefore the flexibility of the metal reducing organisms to adapt to different electron acceptors and donors is much higher.

In addition to the described cytochromes of *Geobacter* and *Shewanella* comparable complexes of other organisms might be able to perform EET via similar mechanisms. The membrane of the Gram-positive acetogen *M. thermoacetica* for example contains b-type cytochromes that could be a possible site of interaction between electrode and organism (Pierce et al., 2008). In fact it could be shown that *M. thermoacetica* together with other cytochrome containing acetogens is able to directly use electrons from a cathode for reduction of CO_2_ (Nevin et al., 2011). Interestingly the same study reports *C. ljungdahlii* is also capable of direct EET even though it is not known to be able to produce any cytochromes (see **Figures 2B** and **3**; Köpke et al., 2010). Could this electron transport rely on other membrane-bound enzymes, or be mediated by an unknown soluble molecule, or enzyme secreted by the organism? Since there is no evidence of such the actual mechanism of EET in *C. ljungdahlii* remains pure speculation to date. However, for other microbes self-excreted redox mediators are indeed an important way to exchange electrons with electrodes in both ways. *P. aeruginosa* for example produces several compounds of different redox potentials such as azurin (E^0′^ = +310 mV), procyanin (E^0′^ = -31 mV), phenazine-1-carbox amide (E^0′^ = –150 mV) and phenazine-1-carboxylic acid (E^0′^ = –275 mV). These enable the organism to adapt to different electron acceptors in the environment, including solid state electrodes (Vijgenboom et al., 1997; Rabaey et al., 2005). A very recent study of Yang et al. (2015) demonstrates that the concentration of such endogenous redox compounds can mediate electron flow in both directions and that the concentration directly correlates with achievable current densities. By heterologous expression of a synthetic flavin biosynthesis pathway from *Bacillus subtilis* in *S. oneidensis* the secreted flavin concentration could be increased ∼26 times, which increased power output as well as inward current of the organism in BES (Yang et al., 2015). Another possibility could be the secretion of whole enzymes that facilitate electron flow from the electrode surface toward organisms. In a very recent study Deutzmann et al. (2015) were the first to report evidence of this mechanism of direct EET by cell-derived free enzymes in a cathodic BES using methanogens. They could show that small surface associated enzymes such as hydrogenases and fumarate reductase were released from *Methanococcus maripaludis* cells and accumulated at the cathode surface where they catalyzed the formation of small mediating molecules such as hydrogen or formate which in turn were immediately taken up by the cells.

The location of the target site of electron transfer decides if an organism might be able to perform direct EET or if a (endogenous or exogenous) mediator is required to transport the charge across the cellular membrane. Additionally each organism can be allocated with a specific redox-potential-window in which its electron transport chains are able to operate, which is also visualized in **Figure 3**. It becomes obvious that compounds of facultative aerobe organisms such as *E. coli* and *S. oneidensis* are represented over a wide range of potentials. This visualizes the high flexibility of both organisms to adapt their electron transport chains to multiple electron donors and acceptors. *G. sulfurreducens* even though a strict anaerobe still covers an impressive range of redox potentials with its many cytochromes and the ability to use soluble metals such as iron and manganese as final electron acceptors. The rnf-complex containing acetogens *C. ljungdahlii* and *A. woodii*, however, are only able to transfer electrons in a limited potential window as they do not contain any cytochromes and are restricted to only one way of respiration at relatively low potentials.

#### Transferring Electrogenic Capabilities between Bacteria

To transfer the ability of one organism that is known to interact with an electrode in a specific way to another organism, synthetic biology tools can be used. It could be shown that expressing enzymes of the Mtr pathway of *S. oneidensis* inside *E. coli* increased current production of the optimized strain (Pitts et al., 2003; Jensen et al., 2010). In 2008 researchers could show that the expression of one single enzyme from *Shewanella’s* Mtr-pathway is enough to transform *E. coli* into a metal respiring bacterium (Gescher et al., 2008). Expression of the cytoplasmic-membrane tetraheme c-type cytochrome CymA enabled the mutant strain to reduce Fe(III) and sustain growth while the native strain lacked this ability. This was independent of the presence of periplasmic or outer-membrane cytochromes for electron transfer. However, it could also be shown that activities of CymA for iron reduction were much lower in complete cells, indicating that diffusion limitations of solid state electron acceptors also play a significant role in this electron transport chain (Gescher et al., 2008). A very important follow up study was able to find evidence for a connection of this transformed electron transport to intracellular carbon flow in *E. coli* (TerAvest et al., 2014). Expression of CymA and Mtr cytochromes from *Shewanella oneidensis* in *E. coli* resulted in a strain that coupled current production to a shift in its metabolic fluxes towards more oxidized products (TerAvest et al., 2014). The possibility to design a target electron flow via artificial complexes across the outer and inner membranes and their connection to cellular redox balance and carbon flow promises many possibilities to design optimized electro active organisms. The above cited example also shows that co-expression of multiple linked enzymes can greatly enhance the electron transfer rates (TerAvest et al., 2014). Therefore the specific electron transport chains of the target organism and their possible connection to the enzymes to be introduced need to be considered when designing electro active bacteria.

### Targeting Electron Transport Impacts the Cellular Energy Metabolism

**FIGURE 4:**
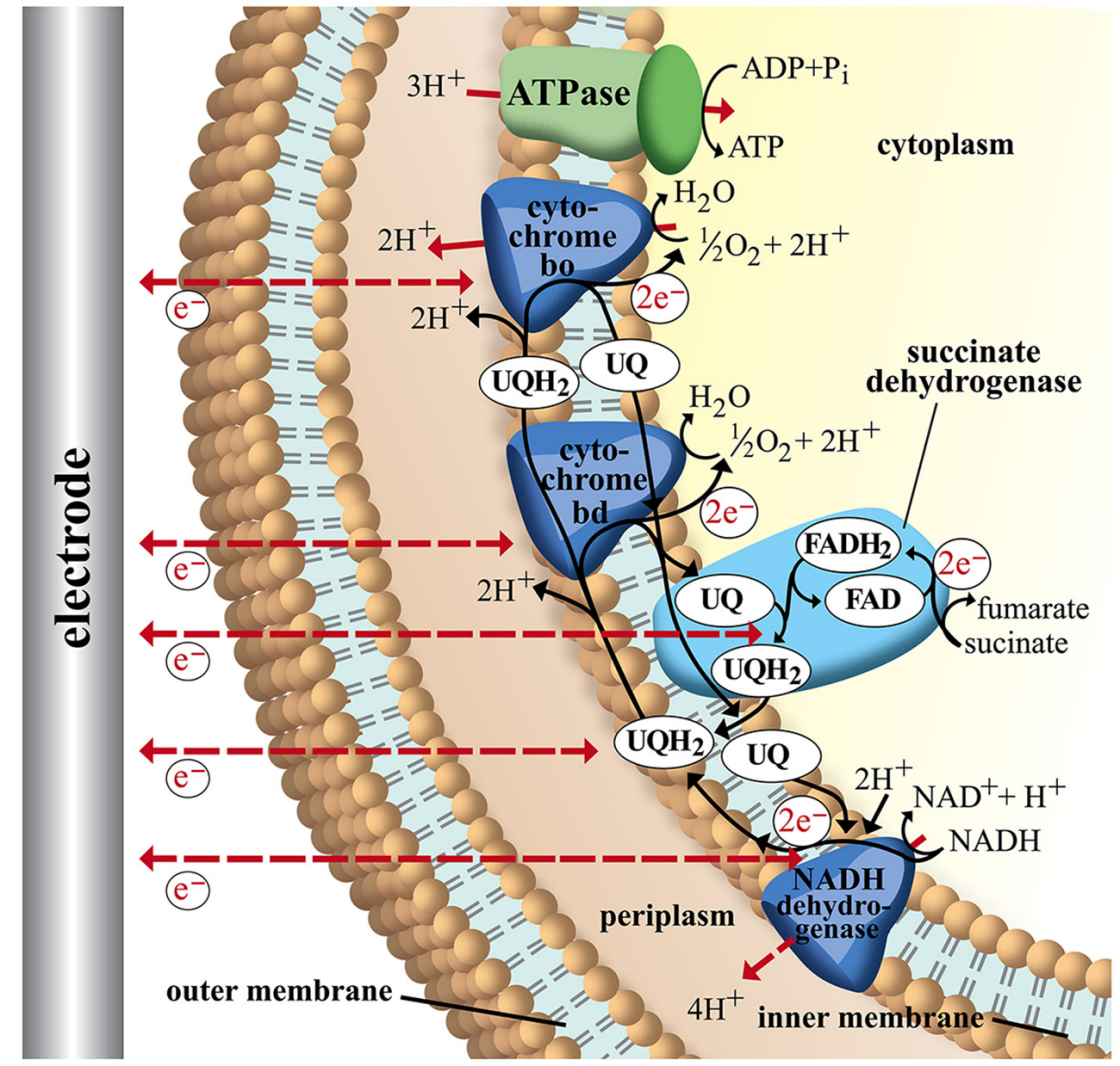
**Schematic image of electron transport chains in *Escherichia coli*.** NADH as electron donor via NADH dehydrogenase (NuoA-N), ubiquinone pool (UQ), succinate-dehydrogenase, and cytochromes *bd* (CydAB) and *bo* (CyoABCD). ATP is generated via F_1_F_0_-ATPase (3H^+^/ATP) from the membrane proton gradient. Possible sites of interaction with an electrode are indicated with dashed arrows. For detailed discussion and references refer to main text.

The achievable energy yield of an electron transport chain is dependent on the difference in electrical potential between electron donor and acceptor. Therefore organisms that are able to respire in multiple ways will always choose available acceptors with the biggest potential difference to the donor (e.g., *E. coli* O_2_ > NO_3_^-^> fumarate). To compare energy efficiencies of different electron transport chains it is referred to the ratio of phosphate to oxygen (P/O quotient), which describes the ratio of mol ATP produced per mol oxygen reduced. The P/O ratio depends on the amount of ions that are transported across the membrane during the corresponding electron-transport chain and are available for ATP-synthesis via gradient-driven ATPase. It is also influenced by the efficiency of the specific ATPase as the amount of ions that are needed for synthesis of one mol ATP differ between organisms (Møller et al., 1996). For example the aerobic electron transport chain of *E. coli* transports up to eight protons across the membrane with NADH as electron donor (2 e^-^) and oxygen as final acceptor (see **Figure 4**; Unden and Bongaerts, 1997). With an ATPase that requires three protons for the conversion of one mol ADP to ATP a P/O ratio of ∼2.7 is calculated (Noguchi et al., 2004).

The ATP yield is an important factor for bacterial growth and can also significantly influence production if the metabolic pathway towards the target compound requires energy. Therefore we present similar to the P/O ratio a hypothetical P/2e^-^ ratio that reflects the ATP yield per pair of electrons transferred between the organism and an electrode in a BES. Some examples are presented in **Table 2**. The electrode can either function as electron donor (cathode) or as final electron acceptor (anode). Since the exact sites of electrode-interaction with the compounds of each electron transport chain remain unknown we assume different scenarios. In column 2 of **Table 2** the assumed site of electrode–bacteria interaction is given together with the corresponding hypothetical reaction. The P/2e^-^ ratio is calculated based on the resulting amount of protons carried across the membrane, which are assumed to be available to drive ATP synthesis via ATPase. An example is explained in detail in the following paragraph.

**Table 2:** Theoretical P/2e^-^ ratios for extracellular electron transfer between electrodes and different sites in the electron transport chains of *E. coli*, *M. thermoacetica* and *C. ljungdahlii*.

Organism	Site of electron transfer	P/2e^-^
**Anodic electron transport**		
*E. coli^a^*	Cytochrome *bo*	2.7
	Cytochrome *bd*	2
	Ubiquinone-pool (UQH_2_ → 2e**^-^** + UQ + 2H^+^_periplasm_)	2
	FAD reduction (FADH_2_ → 2e**^-^** + FAD^+^ + 2H^+^_cytoplasm_)	0.6
	NADH oxidation (NADH → 2e**^-^** + NAD^+^ + 2H^+^_cytoplasm_)	0
**Cathodic electron transfer**		
*E. coli^a^*	NADH reduction (2e**^-^** + NAD^+^ + 2H^+^_cytoplasm_ → NADH)	2.7
	FAD reduction (2e**^-^** + FAD^+^ + 2H^+^_cytoplasm_ → FADH_2_)	1.3
	Ubiquinone reduction (2e**^-^** + UQ + 2H^+^_cytoplasm_ → UQH_2_)	1.3
	Ubiquinone reduction (2e**^-^** + UQ + 2H^+^_periplasm_ → UQH_2_)	0.6
		
*M. thermoacetica^b,c^*	Ferredoxin reduction (2e**^-^** + Fd_ox_ → Fd_red_)	0.75
	Hydrogen evolution (2e**^-^** + 2H^+^_cytoplasm_ → H_2_)	0.375
	NADH reduction (2e**^-^** + NAD^+^ → NADH)	0
		
*C. ljungdahlii^b^*	Ferredoxin reduction (2e**^-^** + Fd_ox_ → Fd_red_)	0.5
	Hydrogen evolution (2e**^-^** + 2H^+^_cytoplasm_ → H_2_)	0.25
	NADH reduction (2e**^-^** + NAD^+^ + 2H^+^_cytoplasm_ → NADH)	0

Column 2 gives the assumed site of electron transfer including hypothetical reaction. For following reactions and references refer to the main text and **Figures 2** and **4**. The corresponding amount of protons transferred across the membrane is assumed to be available for ATP synthesis. P/2e^-^ is mol ATP produced per pair of electrons transferred. ^a^3 H^+^ per ATP in ATPase, ^b^4 H^+^ per ATP in ATPase, ^c^Ech-complex based electron transport chain.

**Figure 4** shows a simplified image of electron transport chains in *E. coli* including possible sites of EET (dashed red arrows). As discussed above it could be shown that cytochromes of *G. sulfurreducens* are able to use electrodes as final electron acceptor. If we assume the cytochromes of *E. coli* are also possible sites of EET we observe that the ATP yield of the electron transport chain depends on the specific cytochrome that is performing the electron transfer to the electrode. In case of cytochrome *bo* transferring electrons to an anode as final electron acceptor 8 H^+^ are transported across the membrane (4 H^+^ from NADH dehydrogenase and 4 H^+^ from UQH_2_ and cytochrome *bo*). With an ATP synthase that requires ∼3 H^+^ per ATP (Noguchi et al., 2004) this leads to a P/2e^-^ ratio of 2.7. Running the electron transport chain with cytochrome *bd* as the final oxidase, however, leads to a P/2e^-^ ratio of 2 as fewer protons are transferred across the membrane (6 H^+^ in total: 4H^+^ from NADH dehydrogenase and 2 H^+^ from cytochrome *bd*). By including soluble redox carriers such as ubiquinones, FAD and NADH to the scenario we can address any site of *E. coli’s* electron transport chain. The observation from this theoretical exercise is to internalize that the achievable ATP yield depends on the actual site of EET. If for example electrons are drawn directly from the cellular NADH pool of *E. coli* no energy gain via chemiosmotic coupling is possible as no proton gradient is established (see **Figure 4**; **Table 2**).

This becomes especially crucial in regards to cathodic electron transfer where processes often aim on microbial metabolism with electrons from electrodes as sole energy and redox source (Rabaey and Rozendal, 2010; Nevin et al., 2011). As discussed in Section “Carbon Respiration of Acetogenic Bacteria” the metabolism of acetogens for example relies on ATP synthesis via membrane-based electron transport complexes. Now we can pick again an example strain and theoretically discuss metabolic impact of EET at different sites. *C. ljungdahlii* does not transfer electrons via cytochromes but was shown to interact with electrodes so we can assume EET via mediators or unknown membrane bound compounds towards its cellular redox compounds. In case electrons from a cathode would lead to hydrogen formation the organism could produce 2 mol of reduced Fd per 4 mol of H_2_ via electron bifurcation in the Hyd-complex (see **Figure 2**; Buckel and Thauer, 2013). The exergonic reaction of reduced Fd and NAD^+^ is used for proton translocation in the rnf complex (2 H^+^/Fd_red_) which in turn enables energy conservation via the membrane bound ATPase. With recent estimates of four protons per mol of ATP a P/2e^-^ ratio of 0.25 is calculated (Schuchmann and Müller, 2014). If it is possible to transfer electrons from a cathode directly to Fd the ratio increases to 0.5 mol ATP per pair of electrons transferred. An electron input toward the NADH pool of the bacterium, however, would not deliver enough energy to establish a proton motive force and therefore ATP generation (see **Table 2**).

Remembering **Figure 3** one can draw the connection between required redox potential and target reaction in a specific electron transport chain of an organism to optimize the conditions for a bio electrochemical process. For a cathodic process of *C. ljungdahlii* for example the applied electrode potential needs to be at least low enough to allow hydrogen formation to enable ATP formation and therefore bacterial growth.

## Conclusion

The remaining question after the presented analysis of microbial electron transport chains is: can we identify an ultimate organism for microbial electrochemical techniques? Requirements for an advantageous organism would be first of all high electro-activity. But depending on the target application different other features can be of crucial importance. Microbial electrosynthesis for example requires a strain that produces industrial relevant products preferably combined with the ability to utilize a variety of cheap substrates. Metabolic engineering tools should be available to optimize production and perform advanced strain designing. For application in bioremediation processes the ability of breaking down a wide range of organic contaminates has priority together of course with efficiency of EET. Other influencing factors are general characteristics that simplify process design such as high oxygen tolerance for anaerobes, no high risk organism, no complicated fermentation conditions such as expensive media supplements, pressurized reactor etc.

The diversity of applications for BES makes it impossible to identify one organism that features all required properties. Still for each application a specific species might outperform others. The highly flexible electron transport chains of metal respiring bacteria such as *Geobacter* and *Shewanella* make them excellent current producers over a wide range of potentials. And acetogens seem to be a very promising group of target organisms for microbial electrosynthesis with their ability to use CO_2_ as sole carbon source. But our study also shows that the complexity of microbial electron transport possibilities bears many challenges for bioelectrochemical techniques. For the successful design of an electrically enhanced bio process the specific electron transport properties of the involved species needs to be considered. Environmental conditions such as applied potentials should be adjusted according to specific target sites for EET. Since these are unknown in most cases an analysis as presented in this manuscript will help to identify best- and worst-case scenarios for microbe-electrode interactions and identification of optimized windows for process parameters. Especially when looking at CO_2_ as a feedstock, the available energy gain through EET will limit the feasibility of many organisms and constrain the list of feasible products. There is currently a lot of interest in microbial electrochemical technologies; however, we believe that without deeper understanding of the underlying electron transfer process development remains a trial and error exercise.

## Author Contributions

FK, IV, and JK designed the study. FK and IV collected the data and drafted the manuscript. FK performed the calculations. All authors edited the manuscript and approved the final version.

## Conflict of Interest Statement

The authors declare that the research was conducted in the absence of any commercial or financial relationships that could be construed as a potential conflict of interest.

## Abbreviations

BES: bio electrochemical system
EET: extracellular electron transport
Fd: ferredoxin
red: reduced
ox: oxidized
